# An instance segmentation dataset of cabbages over the whole growing season for UAV imagery

**DOI:** 10.1016/j.dib.2024.110699

**Published:** 2024-06-29

**Authors:** Yui Yokoyama, Tsutomu Matsui, Takashi S.T. Tanaka

**Affiliations:** a Graduate School of Natural Science and Technology, Gifu University, 1-1 Yanagido, Gifu City 501-1193, JAPAN; b Faculty of Applied Biological Sciences, Gifu University, 1-1 Yanagido, Gifu City 501-1193, JAPAN; c Artificial Intelligence Advanced Research Center, Gifu University, 1-1 Yanagido, Gifu City 501-1193, JAPAN; d Department of Agroecology, Faculty of Technical Sciences, Aarhus University, Forsøgsvej 1, 4200 Slagelse, Denmark

**Keywords:** Annotation, COCO format, Deep learning, Horticulture, Precision agriculture, Remote sensing

## Abstract

Crop growth monitoring is essential for both crop and supply chain management. Conventional manual sampling is not feasible for assessing the spatial variability of crop growth within an entire field or across all fields. Meanwhile, UAV-based remote sensing enables the efficient and nondestructive investigation of crop growth. A variety of crop-specific training image datasets are needed to detect crops from UAV imagery using a deep learning model. Specifically, the training dataset of cabbage is limited. This data article includes annotated cabbage images in the fields to recognize cabbages using machine learning models. This dataset contains 458 images with 17,621 annotated cabbages. Image sizes are approximately 500 to 1000 pixel squares. Since these cabbage images were collected from different cultivars during the whole growing season over the years, deep learning models trained with this dataset will be able to recognize a wide variety of cabbage shapes. In the future, this dataset can be used not only in UAVs but also in land-based robot applications for crop sensing or associated plant-specific management.

Specifications Table

**Table:** 

Subject	Agronomy and Crop Science
Specific subject area	Instance segmentation for cabbage by UAV imagery
Data format	Raw, annotated
Type of data	RGB images, Instance segmentation annotations
Data collection	Cabbage images were collected using UAVs in farmers’ fields over three years (2020, 2021, and 2022). An orthomosaic process was performed using Pix4D and images were split into 500 to 1000 pixels square. Cabbage contours were annotated and exported in a COCO format.
Data source location	Kaizu, Gifu Prefecture, Japan (35°13′N 136°39′E) Yoro, Gifu Prefecture, Japan (35°20′N 136°33′E) Sunomata, Gifu Prefecture, Japan (35°21′N 136°40′E)
Data accessibility	Repository name: Mendeley Data Data identification number: 10.17632/5cp2dyjczk.2 Direct URL to data: https://data.mendeley.com/datasets/5cp2dyjczk/2

## 1 Value of the Data

• This dataset was created by object-based annotation of individual cabbage with laborious manual efforts. The use of distinct dataset and instance segmentation models such as Mask R-CNN [1] and YOLACT [2] enables on-farm assessment of individual cabbage growth (e.g., leaf area index and biomass) by quantifying individual cabbage contours.
• This dataset can also be used for object detection such as YOLO [3] because the JSON file includes the bounding box data if practitioners only need to count the number of cabbages.
• The dataset is highly complementary to the existing dataset [4,5] because it consists of multiple cultivars with different morphological traits, and the images were taken during the whole growing season. Since the image data is classified by cultivar, location and image acquisition time, it is easy to select a specific cultivar or growing season depending on the purpose of model optimisation and validation.
• The dataset can be used for actual crop monitoring. In the future, it is expected to enable automatic plant-level management coupled with land-based agricultural robots.

## 2 Data Description

The dataset includes 458 annotated RGB images and an associated JSON file in COCO format. The JSON file contains 17,621 annotated masks of cabbages in the images. Images are placed in three levels of subfolders. Each subfolder represents the cultivar, location and image acquisition timing (i.e., year and month), respectively. Accordingly, the locations Kaizu, Sunomata and Yoro contain 5455, 6524 and 5642 annotated masks, respectively. The number of annotated masks for cultivars ‘OkinaSP’, ‘Suiryoku’, ‘TCA422’, ‘Yumebutai’ is 7528, 1398, 5376 and 2600, respectively. Meanwhile, cultivars ‘Red cabbage’ and ‘Yumegoromo’ only contain 538 and 181 annotated masks, respectively.

Each image is named in the format “image(a)_(b)_(c)_(d)_(e).png”.

• (a) Image number
• (b) The day the image was taken
• (c) Location
• (d) Cultivar name
• (e) Image size

**Fig. 1:**
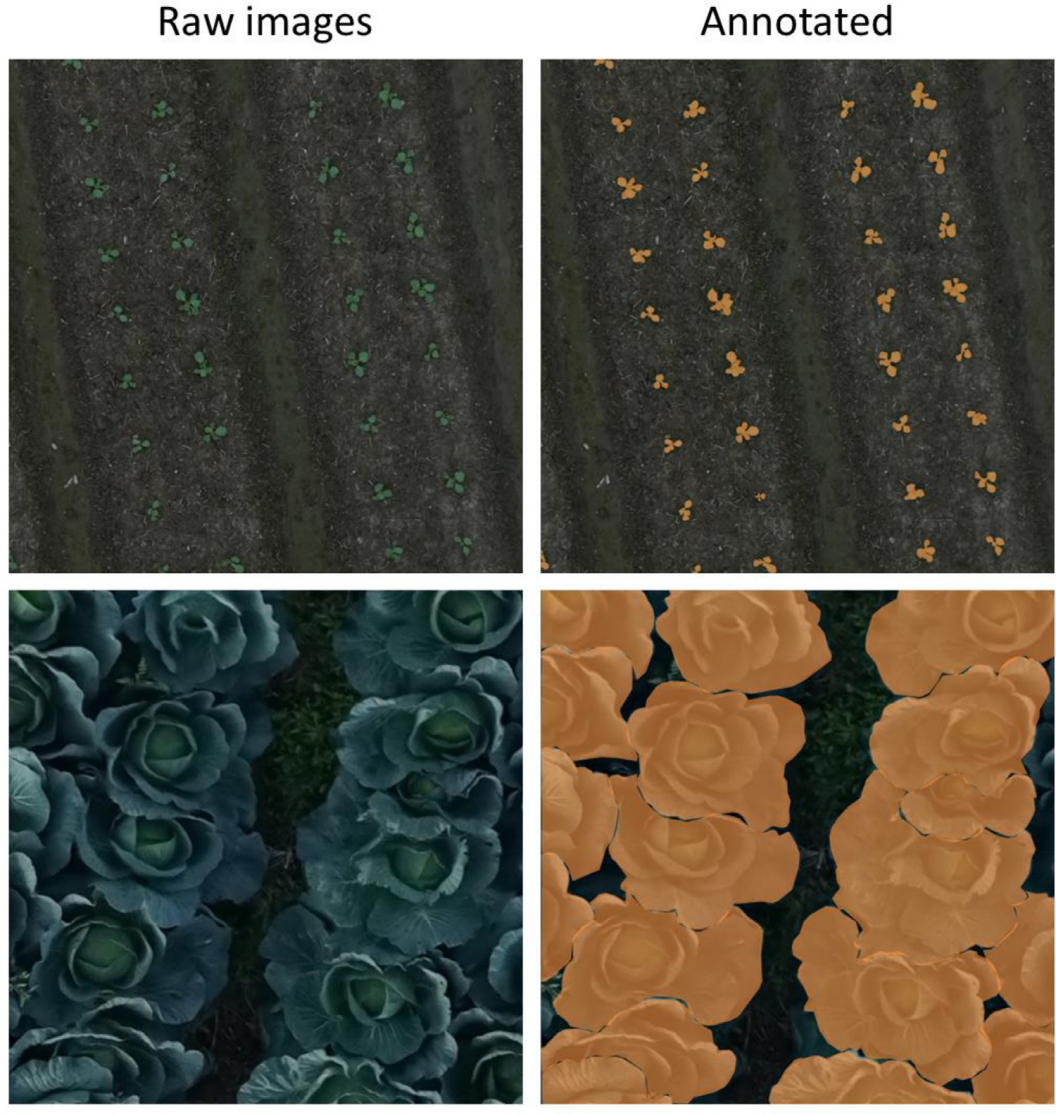
Raw and annotated images of the dataset.

All images have annotated information in a JSON file “annotated.json”. Fig. 1 shows the segmentation masks of the dataset. Each cabbage mask was enclosed along the outline and saved as the label name “cabbage”.

## 2 Experimental Design, Materials and Methods

### 2.1 Field data collection

The RGB images of cabbages were collected in 2020, 2021, and 2022 in farmers’ fields in three cities, including Yoro, Sunomata and Kaizu, Gifu Prefecture, Japan. Table 1 shows the cultivars grown in each city and transplanting day. In Kaizu on 2020 and 2021, cabbages were cultivated by transplanting one row in a ridge. Others were cultivated by transplanting two rows in a ridge. This also makes images look different due to the different plant density and transplant spacing. Fertilisation and pest/disease management was performed according to the recommendation suggested by the local crop advisory service. All the cabbages were grown under rainfed conditions. During the cabbage growing season from September to December, RGB images were taken using a UAV. All images were captured between 9:00 and 16:00 under various cloud conditions from clear sky to thick clouds. Given the temporally varying light intensity even within a day and varying sun angles over the growing seasons from summer to winter, entire dataset covers the effect of a wide range of varying illumination conditions on image quality. In 2020, Phantom4 (DJI, Shenzhen, China) was used for image acquisition at a 20 m altitude with 75% front and side overlap. In 2021 and 2022, the digital camera α6600 (Sony, Tokyo, Japan) mounted on MATRICE300 (DJI, Shenzhen, China) was used for image acquisition at a 30 m altitude with 70–80% front and side overlap. The coordinates of RGB images were measured using KlauPPK (Klau Geomatics, New South Wales, Australia) with a 0.03-m accuracy.

**Table 1:** Transplant day of each day and cultivar.

Year	City	Cultivar	Transplant Day
2020	Kaizu	OkinaSP	Aug 30, 2020
2021	Yoro Sunomata Kaizu	OkinaSP Suiryoku Yumebutai OkinaSP TCA422 OkinaSP	Aug 30, 2021 Aug 30, 2021 Aug 30, 2021 Sep 1, 2021 Sep 2, 2021 Aug 30, 2021
2022	Yoro Sunomata	OkinaSP Suiryoku Yumebutai Red cabbage OkinaSP TCA422 Yumegoromo	Aug 29, 2022 Aug 29, 2022 Aug 29, 2022 Aug 29, 2022 Aug 29 and 30, 2022 Aug 29 and 30, 2022 Aug 29 and 30, 2022

### 2.2 Data preprocessing

The orthomosaic process was performed using Pix4D mapper version 4.6.4 (Pix4D, Prilly, Switzerland) based on the processing template 3D Maps. The orthomosaic images were split into 515–1000 pixel squares using GDAL as a Python module. The spatial resolution of the resultant split images ranged from 3.3 to 6.3 mm. A total of 458 split images were randomly selected from the split images. The cabbage masks were manually annotated using the COCO annotator [6]. The drawing tablet Cintiq 16 (Wacom Co., Ltd, Saitama, Japan) was used for the accurate and efficient annotations. To accurately draw the semantic annotations for the individual cabbage, the application of expert judgement derived from experiences in field survey becomes indispensable particularly in situations involving the overlapping of multiple cabbages. Therefore, highly trained technician was involved in the annotation process, and every annotation were carefully checked by the authors.

## Limitations

None.

## Ethics Statement

The study does not involve experiments on humans or animals.

## CRediT authorship contribution statement

**Yui Yokoyama:** Conceptualization, Methodology, Data curation, Writing – original draft. **Tsutomu Matsui:** Writing – review & editing, Supervision. **Takashi S.T. Tanaka:** Conceptualization, Methodology, Data curation, Writing – review & editing, Supervision.

## Data Availability

An annotated image dataset of cabbages for instance segmentation (Original data) (Mendeley Data)
